# Cleanifier: contamination removal from microbial sequences using spaced seeds of a human pangenome index

**DOI:** 10.1093/bioinformatics/btaf632

**Published:** 2025-11-18

**Authors:** Jens Zentgraf, Johanna Elena Schmitz, Sven Rahmann

**Affiliations:** Algorithmic Bioinformatics, Saarland University, Saarbrücken 66123, Germany; Saarbrücken Graduate School of Computer Science, Saarland Informatics Campus, Saarbrücken 66123, Germany; Center for Bioinformatics Saar, Saarland Informatics Campus, Saarbrücken 66123, Germany; Algorithmic Bioinformatics, Saarland University, Saarbrücken 66123, Germany; Saarbrücken Graduate School of Computer Science, Saarland Informatics Campus, Saarbrücken 66123, Germany; Center for Bioinformatics Saar, Saarland Informatics Campus, Saarbrücken 66123, Germany; Algorithmic Bioinformatics, Saarland University, Saarbrücken 66123, Germany; Center for Bioinformatics Saar, Saarland Informatics Campus, Saarbrücken 66123, Germany

## Abstract

**Motivation:**

The first step when working with DNA data of human-derived microbiomes is to remove human contamination for two reasons. First, many countries have strict privacy and data protection guidelines for human sequence data, so microbiome data containing partly human data cannot be easily further processed or published. Second, human contamination may cause problems in downstream analysis, such as metagenomic binning or genome assembly. For large-scale metagenomics projects, fast and accurate removal of human contamination is therefore critical.

**Results:**

We introduce Cleanifier, a fast and memory frugal alignment-free tool for detecting and removing human contamination based on gapped *k*-mers, or spaced seeds. Cleanifier uses a pangenome index of known human gapped *k*-mers, and the creation and use of alternative references is also possible. Reads are classified and filtered according to their gapped *k*-mer content. Cleanifier supports two filtering modes: one that queries all gapped *k*-mers and one that queries only a sample of them. A comparison of Cleanifier with other state-of-the-art tools shows that the sampling mode makes Cleanifier the fastest method with comparable accuracy. When using a probabilistic Cuckoo filter to store the complete *k*-mer set, Cleanifier has similar memory requirements to methods that use a sampled minimizer index. At the same time, Cleanifier is more flexible, because it can use different sampling methods on the same index.

**Availability and implementation:**

Cleanifier is available via gitlab (https://gitlab.com/rahmannlab/cleanifier), PyPi (https://pypi.org/project/cleanifier/), and Bioconda (https://anaconda.org/bioconda/cleanifier). The pre-computed human pangenome index is available at Zenodo (https://doi.org/10.5281/zenodo.15639519).

## 1 Introduction

Removing host DNA contamination is a crucial initial step in metagenomic analyses for two main reasons. First, microbiome samples derived from a human host inevitably contain human DNA that can reveal private personal information. Recent research highlights the importance of accurate and complete removal of human DNA (Tomofuji *et al.* 2023, Guccione *et al.* 2025). Due to these privacy concerns, many public databases, such as the NCBI Sequence Read Archive (SRA) (Kodama *et al.* 2012), require that human-derived metagenomic sequence data is free of human DNA prior to publishing. Second, Pereira-Marques *et al.* (2019) showed that increasing proportions of host DNA can deteriorate the sensitivity of detecting low abundant species during taxonomic profiling.

Given the ever-growing amount of microbiome data (Proctor *et al.* 2019), there is an unmet demand for a host filtering tool that is simultaneously accurate, easy-to-use, fast, and has low memory requirements.

### 1.1 Contributions

We introduce Cleanifier, a rapid and precise alignment-free method to remove host contamination. Cleanifier builds a comprehensive database of gapped *k*-mers (spaced seeds), encompassing the human T2T reference genome (Nurk *et al.* 2022), all 47 genome assemblies from the Human Pangenome Reference Consortium (Liao *et al.* 2023), common variants from the 1000 Genome Project (Auton *et al.* 2015), all HLA gene variants from the IPD-IMGT/HLA database (Barker *et al.* 2023) and human cDNA (Dyer *et al.* 2025), thus containing most of the known variation in the human pangenome. Using the pre-built human index, users only need to perform the fast filtering step using a simple command-line tool supporting single-end, paired-end, short and long read sequencing data, both in compressed and uncompressed FASTQ format. In addition, Cleanifier features a sampling mode that makes it the fastest method among state-of-the-art tools.

### 1.2 Related previous work

Existing tools include both alignment-based and alignment-free *k*-mer-based methods, as summarized in Table 1. Alignment-based tools use aligners, such as Bowtie2 (Langmead and Salzberg 2012), Minimap2 (Li 2018), or BWA (Li and Durbin 2009), to remove all reads that map and align well to the human reference. For example, KneadData (https://huttenhower.sph.harvard.edu/kneaddata/) uses Bowtie2, and Hostile (Constantinides *et al.* 2023) uses Bowtie2 for short reads and Minimap2 for long reads. To increase mapping sensitivity, Hostile provides a custom index containing the human T2T genome and all HLA gene variants from the IPD-IMGT/HLA database (Barker *et al.* 2023). To avoid the slow and computationally expensive alignment step, *k*-mer-based methods are an alternative with a similar accuracy. The taxonomic classifiers Kraken 2 (Wood *et al.* 2019) and KrakenUniq (Breitwieser *et al.* 2018) can be used to classify the reads and to then remove all reads classified as human. Other *k*-mer-based tools are the Human Read Removal Tool (HRRT), which is based on the Sequence Taxonomic Analysis Tool (STAT) (Katz *et al.* 2021), and Deacon (Constantinides *et al.* 2025), which uses a human minimizer database created from the assemblies from the Human Pangenome Reference Consortium, the T2T reference and the genome assembly GRCh38.p14 (Nurk *et al.* 2022, Liao *et al.* 2023).

**Table 1.** Overview of tools for human contamination removal and their supported input data.^a^

Tool	Ref.	Single-end	Paired-end	Short reads	Long reads	Compression	Alt. host/custom
Hostile	Constantinides *et al.* (2023)	✓	Separate	✓	✓	✓	✓
noHuman	Hall and Coin (2024)	✓	Separate	✓	✓	✓	✘
Kraken2	Wood *et al.* (2019)	✓	Separate	✓	✓	Input only	✓
+Krakentools	Lu *et al.* (2022)						
HRRT	Katz *et al.* (2021)	✓	Interleaved	✓	✓	✘	✘
Deacon	Constantinides *et al.* (2025)	✓	Both	✓	✓	✓	✓
Cleanifier	This work	✓	Separate	✓	✓	✓	✓

a Paired-end data have to be provided either as two separate files or as interleaved paired-end format. The standard Kraken 2 database may be used to remove sequences from any host, and Cleanifier may be used to remove sequences from other hosts by building a custom host genome or pangenome database.

### 1.3 Outline

In Section 2, we describe the data structures used for indexing gapped *k*-mers and how we query them during the filtering step of Cleanifier. In Section 3, we compare Cleanifier with Hostile (alignment-based), Kraken 2 (*k*-mer-based taxonomic classifier), noHuman (Kraken 2 wrapper with custom database), HRRT (*k*-mer-based), and Deacon (minimizer-based). We give some concluding remarks in Section 4.

## 2 Materials and methods

Before we describe the method in detail, we introduce basic definitions. We consider nucleotide sequences over the DNA or RNA alphabet Σ. Since the alphabet size is |Σ|=4 in both cases, ${\;\text{log}\;}_{2}|\Sigma |=2$ bits encode a single nucleotide.

A *k-mer* is a sequence of length *k* over Σ, i.e. an element of $\Sigma^{k}$. For a sequence $s\in \Sigma^{*}$ with |s|≥k (i.e. of any length at least *k*), a substring of length *k* is called a *k-mer of s*. A *k*-mer *x* can be encoded bijectively as a 2*k*-bit integer enc(x).

Since DNA is double-stranded, the reverse complement of a DNA *k*-mer and the *k*-mer itself represent the same molecule and should be treated as equivalent. To represent a *k*-mer *x*, we integer-encode both *x* and its reverse complement rc(x) and then take the larger integer to represent both. Thus, the canonical code of a *k*-mer *x* is cc(x):=max{enc(x),enc(rc(x))}.

Example 1 Each nucleotide is encoded as a base-4 digit by two bits ($A={\left(0\right)}_{4}={\left(00\right)}_{2}$, $C={\left(1\right)}_{4}={\left(01\right)}_{2}$, $T={\left(2\right)}_{4}={\left(10\right)}_{2}$, and $G={\left(3\right)}_{4}={\left(11\right)}_{2}$). The encoding enc(x) for a *k*-mer *x* reads *x* as a base-4 number.

sequence *s*=TACGCGTAAG; using *k* = 5

k-mer x     enc(x)  rc(x)  enc(rc(x))  cc(x)

TACGC     541 GCGTA 888    888

ACGCG     119 CGCGT 478    478

 CGCGT    478 ACGCG 119    478

  GCGTA   888 TACGC 541    888

   CGTAA  480 TTACG 647    647

    GTAAG 899 CTTAC 417    899

A *k*-mer is a contiguous substring of length *k*. Hence, a single variant (or sequencing error) in a read changes *k* consecutive *k*-mers. To be more tolerant against errors, previous alignment-free methods proposed to use gapped *k*-mers, also called spaced seeds, that consider *k* significant positions in a window of length *w* (Břinda *et al.* 2015, Wood *et al.* 2019, Zentgraf and Rahmann 2022). Exactly which *k* out of the *w* positions are considered is specified by a mask defined as follows.

Given two integers w≥k≥2, a (k,w)-*mask* is a string μ of length *w* over the alphabet {#,_} that contains exactly *k* times the character # and w−k times the character _. The positions marked # are called *significant*, and the positions marked _ are called *insignificant*. We call *k* the *weight* of the mask and *w* its *width* or *window length*. The pair (k,w) is called the *shape* of the mask. A mask μ may also be represented as the tuple κ of significant positions: $\kappa =\{j:\;0\leq j<w\;\text{and}\;\mu_{j}=\#\}$.

We require that the first and last position of a mask are significant. We only consider symmetric masks to obtain the same canonical integer encodings for the forward and reverse strands. Since the robustness against substitutions depends on the positions of the significant and insignificant positions, we only consider masks with good error properties (Zentgraf and Rahmann 2025).

Example 2 Given the same sequence as in the previous example and the (5, 7)-mask ##_#_##, we extract the following canonical gapped *k*-mer codes, where only the nucleotides in the significant positions are considered.

sequence s=TACGCGTAAG; using (5,7)-mask ##_#_##

seed x       enc(x)  rc(x)   enc(rc(x))  cc(x)

TA_G_GT    574  AC_C_TA 88   574

AC_C_TA    88  TA_G_GT 574   574

 CG_G_AA   496  TT_C_CG 663   663

  GC_C_AG  851  CT_G_GC 445   851

### 2.1 Index data structures: Cuckoo hash tables and filters

Cleanifier supports both an exact multiway bucketed Cuckoo hash table and a probabilistic windowed Cuckoo filter for storing the pangenomic gapped *k*-mers. These data structures were chosen because of their low memory consumption, fast lookup times and their support of online insertions, i.e. it is not necessary to know the set *S* of stored keys in advance; a size estimate of |*S*| suffices (Zentgraf and Rahmann 2022).

#### 2.1.1 Multi-way bucketed Cuckoo hash table

Hash tables are data structures that store a set *S* of keys from a universe U⊃S exactly and answer membership queries or retrieve stored information (a value) associated with each key x∈S. In (d,ℓ) bucketed Cuckoo hashing, an array with *n* slots is divided into b=⌈n/ℓ⌉ buckets with ℓ slots each. To store a key x∈U, we use d≥2 hash functions $f_{1},\ldots ,f_{d}:U\to [b]:=\{0,1,\ldots ,b-1\}$ to compute *d* possible bucket addresses, where a key *x* may be stored (together with its optional value). Hence, *x* may be stored in any of the dℓ slots at *d* distinct memory locations. When checking the presence of a key, all dℓ slots are searched, until either the key or a free slot is found. Since all slots of one bucket are often contained in the same cache line, we generally only have up to *d* cache misses per lookup, and for less full tables frequently only a single cache miss, yielding very fast lookup times.

The insertion of a key *x* works as follows: If we find an empty slot in one of the buckets given by $f_{1}(x),\ldots ,f_{d}(x)$, the key is inserted in the first free slot. If no free slot is found, we remove a random key from one of the dℓ slots and try to re-insert it in one of its alternative buckets. This step is repeated until either a free slot is found or a given maximum number of steps is reached. This constant threshold of steps guarantees that insertions are done in constant time, or fail. If an insertion fails, we create a larger hash table if the table is full, or we use different hash functions and redo the insertion for all elements. In the common configuration of (3,4) bucketed Cuckoo hashing, the maximum load (fraction of non-empty slots) before insertions fail with high probability of ≈98%, leading to a low memory overhead (Walzer 2023).

#### 2.1.2 Probabilistic windowed Cuckoo filter

Probabilistic filters are space efficient data structures to answer membership queries with a certain false positive probability. If we query a stored key x∈S, the filter always correctly reports the key as present. However, if we query a key x∉S, the filter may erroneously report the key as present with a small controllable error probability ε, called false positive rate (FPR). The FPR is closely linked to the space requirements of the filter, i.e. the required space increases for lower FPRs. Cuckoo filters use the same collision resolution strategy as Cuckoo hash tables and thus have the same advantages as Cuckoo hash tables, but with the additional benefit of requiring less space due to storing only a small *p*-bit fingerprint instead of an exact representation of the key *x*. Since we compare a *p*-bit fingerprint with all fingerprints stored in the dℓ possible slots, the probability to falsely report a key as present is bounded by $2^{-p}\cdot d\ell$.

The (d,ℓ) windowed Cuckoo filters work similar to a bucketed Cuckoo filter, but the *n* slots are divided into n−ℓ+1 overlapping windows instead of buckets. At each slot, a new window starts and overlaps with the next ℓ−1 windows. The windowed layout has been proven to admit higher load thresholds than the bucketed layout for the same (d,ℓ) values (Walzer 2023, Schmitz *et al.* 2025). We here use (2,2) windowed Cuckoo filters at 95% load, using 16 bits per slot for an FPR of 2^−14^ < 1/16 000, which results in approximately 16.8=16/0.95 bits per key overall.

### 2.2 Indexing the human pangenome

During the indexing step, all gapped *k*-mers of the host genome are inserted into the index using a specified symmetric (k,w) mask. More precisely, the index stores canonical codes of the gapped *k*-mers present in the provided reference sequences. The index data structure is either a bucketed Cuckoo hash table (with exact canonical codes) or a windowed Cuckoo filter (with fingerprints), as described above. The advantage of the Cuckoo filter is that it requires less space compared to the Cuckoo hash table, at the cost of a small FPR, which we show in Section 3 to be negligible in practice.

We provide a pre-computed pangenome index for filtering human reads using the following (29,33) symmetric gapped *k*-mer mask ’######_#######_###_#######_######’. It guarantees at least 50 out of 100 covered positions for every substring of length 100 for which there exists a substring of the genome that differs by at most three substitutions (Zentgraf and Rahmann 2025). The index is based on the T2T reference genome (Nurk *et al.* 2022), which provides the benefit that it does not contain undefined bases. By using gapped *k*-mers, the approach is already robust against SNPs, which is further improved by including the variants from the 1000 Genome Project (Auton *et al.* 2015). From the VCF files of all 3202 genomes, we extract all variants, namely substitutions, insertions, and deletions, with an allele frequency of at least 1%. We replace the region in the reference with the provided alternative sequence and add all gapped *k*-mers ±50 bp of the new sequence to the index. In addition, we include the gapped *k*-mers from all 47 pangenome assemblies of the Human Pangenome Reference Consortium (Liao *et al.* 2023). Since the major histocompatibility complex (MHC) is a highly variable region in the genome, we further add all the human leukocyte antigen (HLA) gene variants stored in the IPD-IMGT/HLA database (Barker *et al.* 2023) to the index. To be able to remove human contamination from RNA-seq data, we additionally add all gapped *k*-mers from the human cDNA transcripts in the Ensembl database (Dyer *et al.* 2025).

A comparison concerning classification accuracy between this pangenome index and an index that only contains the gapped *k*-mers of the T2T reference genome is provided in the Supplement, available as supplementary data at *Bioinformatics* online.

### 2.3 Filtering microbiome samples

To decide whether a read *s* belongs to the host organism or not, we query the gapped *k*-mers of *s* in the index. If we find a gapped *k*-mer in the index, we label the base pairs overlapping with this gapped *k*-mer as *covered*. Given a threshold T∈[0,1], a read is then classified as originating from the host genome if at least *T*|*s*| base pairs are covered.

Cleanifier supports two classification modes, a faster sampling mode and a slower sensitive mode:

- In *sensitive* mode, all gapped *k*-mers of the read are queried. If a gapped *k*-mer exists in the index, the significant positions in the read are marked as covered.
- In *sampling* mode, for a mask of shape (k,w), we query the first gapped *k*-mer of the read and then skip the next ⌊w/2⌋ consecutive gapped *k*-mers before we query the next one, and so on. Hence, every base pair has a chance of being covered by two distinct gapped *k*-mers. If a gapped *k*-mer is present in the index, we count all *w* positions (not only the significant ones) as covered.

The above holds for single-end reads. To process paired-end reads, each read of a pair is first classified independently. If at least one of the two ends is classified as a host read, the whole pair is removed.

### 2.4 Implementation details

Cleanifier is implemented as a workflow-friendly command-line tool in Python, using the numba package (Lam *et al.* 2015) for just-in-time compiling the Python code. We use our own implementations of windowed Cuckoo filters (Schmitz *et al.* 2025) and bucketed Cuckoo hash tables (Zentgraf and Rahmann 2022) that use double quotienting for storing partial keys (without sacrificing exactness) to reduce the space requirements of the hash table.

#### 2.4.1 Parallelization

Our implementation parallelizes both the indexing and the filtering step of Cleanifier using a consumer–producer architecture. For indexing, parallelization is over sub-tables, with a single thread being responsible for inserting keys or fingerprints into a sub-table. An additional outer hash function selects the sub-table for each *k*-mer and delegates insertion to the corresponding inserter thread. The threads communicate over lock-free buffers that use atomic volatile ready_to_read and ready_to_write flags. For filtering with its read-only workload, parallelization is trivial over chunks of reads or read pairs.

#### 2.4.2 I/O and consumer–producer architecture

For indexing, a dedicated I/O thread reads (chunks of) the genomic input sequences into buffers that are consumed by threads that split the reads into their gapped *k*-mers and write their canonical codes into sub-table-specific buffers. Inserter threads (one per sub-table) consume these buffers and perform the insertions.

For filtering, a dedicated I/O thread reads the FASTQ files into multiple buffers. Compressed input and output are supported via external tools in separate processes with data being transferred via pipes. For paired-end data, each input buffer is divided into two halves, the first half contains the reads of the first pair and the second half of the other pair. The classifier threads consume the buffers from the I/O threads, classify the reads by querying the gapped *k*-mers and write the classification results into their respective output buffers. A writer thread consumes the output buffers from the classifier threads and writes the reads that are not classified as human to the output FASTQ files.

#### 2.4.3 Index in shared memory

Cleanifier provides the option to load the index into shared memory once, where multiple independent Cleanifier instances may use it simultaneously for (read-only) filtering. This has two advantages in many-sample scenarios. First, it saves the time for loading the index into memory each time a Cleanifier process is started. Second, it enables parallelization over multiple instances without increasing the memory footprint.

## 3 Results

First, we describe the human and microbiome datasets and the tools used in our evaluation. We then compare all tools with respect to their filtering accuracy, running time, and memory requirements on short and long read sequencing data. In the Supplement, available as supplementary data at *Bioinformatics* online, we show a comparison between tools when using custom indexes that contain only the human T2T reference genome.

Benchmarks were run on an Ubuntu (24.04 LTS) server with two AMD EPYC 9534 64-Core Processors, 1.5 TB of 4800-MHz DDR5 memory and a KIOXIA CD8P SSD. We measured running times and maximum memory usage with /usr/bin/time -v.

### 3.1 Datasets

We used human and microbiome datasets as detailed below. Dataset URLs are provided in the Supplement, available as supplementary data at *Bioinformatics* online.

#### 3.1.1 Human data

We applied the tools to human whole genome sequencing datasets provided by the Genome in a Bottle (GIAB) consortium (Zook *et al.* 2016), HG002 to HG006. In principle, these datasets should contain only human reads, but some contamination with viruses or bacteria or technical artifacts cannot be excluded. It is therefore unclear whether we should expect that 100% of all reads should be removed. We evaluated the performance for short reads on paired-end Illumina data, downsampled to contain 50 million 2 × 125 bp read pairs from each dataset HG002–HG006. For long reads, we used Pacific Biosciences (PacBio) reads of HG002, downsampled to 5 million reads.

#### 3.1.2 Microbiome data

We downloaded the CAMI 2 challenge human microbiome dataset (Meyer *et al.* 2022, Fritz *et al.* 2021) for five different body sites (gastrointestinal, airways, oral, skin, and urogenital) as a gold standard that contains only microbial reads. The dataset is a mixture of reads from different species based on the microbiome profiles from the Human Microbiome Project (Huttenhower *et al.* 2012). We combined the available FASTQ files, such that each file per body site contains 50 million paired-end Illumina short reads (2 × 150 bp), or 5 million PacBio long reads, respectively.

### 3.2 Tools

We evaluated Cleanifier against five state-of-the-art methods for human contamination removal: Hostile, Kraken 2 with Krakentools, noHuman (based on Kraken 2), HRRT, and the recent Deacon. We ran all tools using eight threads and their respective default parameters, unless explicitly stated otherwise. We provide a brief description of the evaluated tools below.

We used Cleanifier (version 1.1.3) with the human pangenome index described above and a coverage threshold of 0.5 (the default) for classification. We ran four different Cleanifier versions, combining either the exact hash table or the probabilistic filter with an FPR of $2^{-14}$ with the sensitive mode or the sampling mode for classification.

Hostile (version 2.0.0) is an alignment-based approach to remove human contamination by aligning all reads to the human genome using Bowtie2 for short reads and Minimap2 for long reads (Constantinides *et al.* 2023). We ran Hostile with its default index comprised of the human T2T reference (Nurk *et al.* 2022) and HLA variants from the IPD-IMGT/HLA database (Barker *et al.* 2023).

Kraken 2 (version 2.1.3) is a fast *k*-mer-based taxonomic classification tool for metagenomic sequencing data (Wood *et al.* 2019). We evaluated Kraken 2 using the standard database (available for download at https://benlangmead.github.io/aws-indexes/k2). After taxonomic classification, reads that are not classified as *Homo sapiens* (taxonomy ID: 9606) are extracted using Krakentools (Lu *et al.* 2022).

noHuman (version 0.3.0) is a Kraken 2 wrapper specifically built to remove human contamination (Hall and Coin 2024). It uses a custom Kraken database built from all genomes available in the Human Pangenome Reference Consortium (Liao *et al.* 2023).

HRRT (commit 70ac740) is part of the SRA Taxonomy Analysis Tool STAT (Katz *et al.* 2021). Reads are removed by querying *k*-mers in a MinHash-based index that contains all *k*-mers present in human-derived eukaryotic species; excluding all *k*-mers that are known to also occur in non-eukaryotic species.

Deacon (version 0.6.0, under active development) builds a pangenome index that contains all human minimizers using a fast SIMD minimizer construction (Groot Koerkamp and Martayan 2025). Reads are classified as human if at least two minimizers are found in the index (Constantinides *et al.* 2025). We use the pre-built panhuman-1 index (available at https://github.com/bede/deacon).

### 3.3 Results on short reads

**Figure 1.**
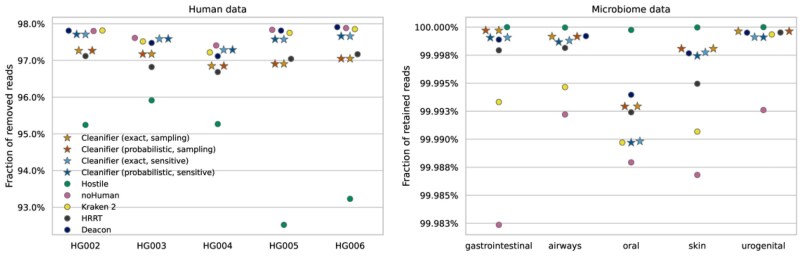
Results on Illumina paired-end short reads for human datasets (left panel; fraction of removed reads) and microbiome data (right panel; fraction of retained reads); higher is better. The different Cleanifier versions are indicated by differently colored stars; other tools by circles.

We report the fraction of correctly classified reads (i.e. correctly removed reads from the human datasets, and correctly retained reads in the microbial datasets) in Fig. 1. We note that no tool removes more than 98% of the human reads. This does not necessarily mean that all tools miss at least 2% of the reads. All of these five whole genome sequencing datasets (HG002–HG006) may contain non-human contamination (e.g. Epstein–Barr virus, phiX phage), or technical artifacts. The correct set of reads to be removed is unknown, and probably not 100%, but we still may assume that more removed human reads are better. On the other hand, 100% of retained microbial reads from the CAMI datasets are certainly desirable, and almost all tools achieve close to 100% retention rate, with the lowest value of any tool on any dataset being 99.983%.

Kraken 2 and noHuman classify many reads as possibly human, resulting in the highest removal rate on human data, but also in the lowest retention rate on microbiome data.

In contrast, Hostile has the best microbiome retention among the tools, but it is much less sensitive to human reads, to a degree where it may be considered unsafe to use in privacy-sensitive settings. While recent studies have shown that small quantities of human reads can expose patient-identifying information (Guccione *et al.* 2025), a more thorough evaluation of the retained human reads is required to ascertain if they contain privacy-exposing information (e.g. regions with many known SNPs).

All Cleanifier versions, Deacon and HRRT have a high accuracy on both human and microbiome data, with Cleanifier having a slightly higher accuracy compared to HRRT, and Deacon performing slightly better on human sequence removal.

Among the Cleanifier versions, the exact and probabilistic data structures have almost the same accuracy on all samples, showing that for the default coverage threshold of 0.5, the small FPR incurred by the filter for a single *k*-mer query is negligible in practice, as several *k*-mer matches are required to classify a read as human. The sampling mode (red/orange stars) of Cleanifier is slightly less sensitive to human reads compared to the sensitive mode (blue/cyan stars): On average, 0.51% of all reads are additionally retained in the human datasets. On microbiome data, the sampling and sensitive mode have a comparable accuracy.

To investigate the question which reads from the human datasets may be in fact of non-human origin, we created Venn diagrams for the retained human reads for Cleanifier (probabilistic) in both sampling and sensitive mode and each other tool, based on HG002 (Fig. 3). More detailed comparisons can be found in the Supplement, available as supplementary data at *Bioinformatics* online. The reads classified as non-human by Cleanifier (in both sampling and sensitive mode) are almost a true subset of the reads retained by Hostile and share a high overlap with noHuman, Kraken, HRRT, and Deacon, suggesting that those reads may indeed be contaminants or other technical artifacts.

### 3.4 Running time on short reads

**Figure 2.**
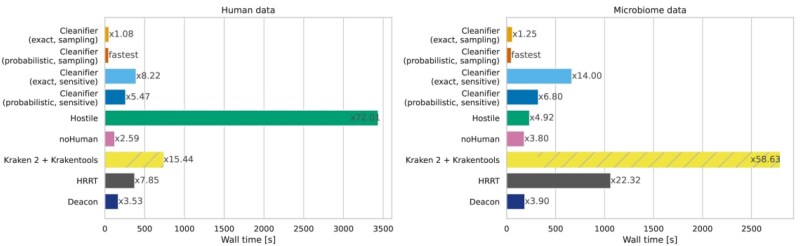
Running time in seconds on Illumina short read datasets with 50 million paired-end reads (left panel: human data; right panel: microbiome data). All tools are run with eight threads. The running time is averaged over five human and microbiome datasets, respectively. Factors next to bars show running times relative to the fastest tool (Cleanifier in sampling mode using a probabilistic Cuckoo filter).

We measured wall clock running times for reading, classifying, and writing uncompressed FASTQ files and configured all tools to output only the microbial reads. Figure 2 shows these times for FASTQ files with 50 million paired-end short reads using eight threads, separately for human and microbial reads. Speedup factors for using Cleanifier with different numbers of threads can be found in the Supplement, available as supplementary data at *Bioinformatics* online.

**Figure 3.**
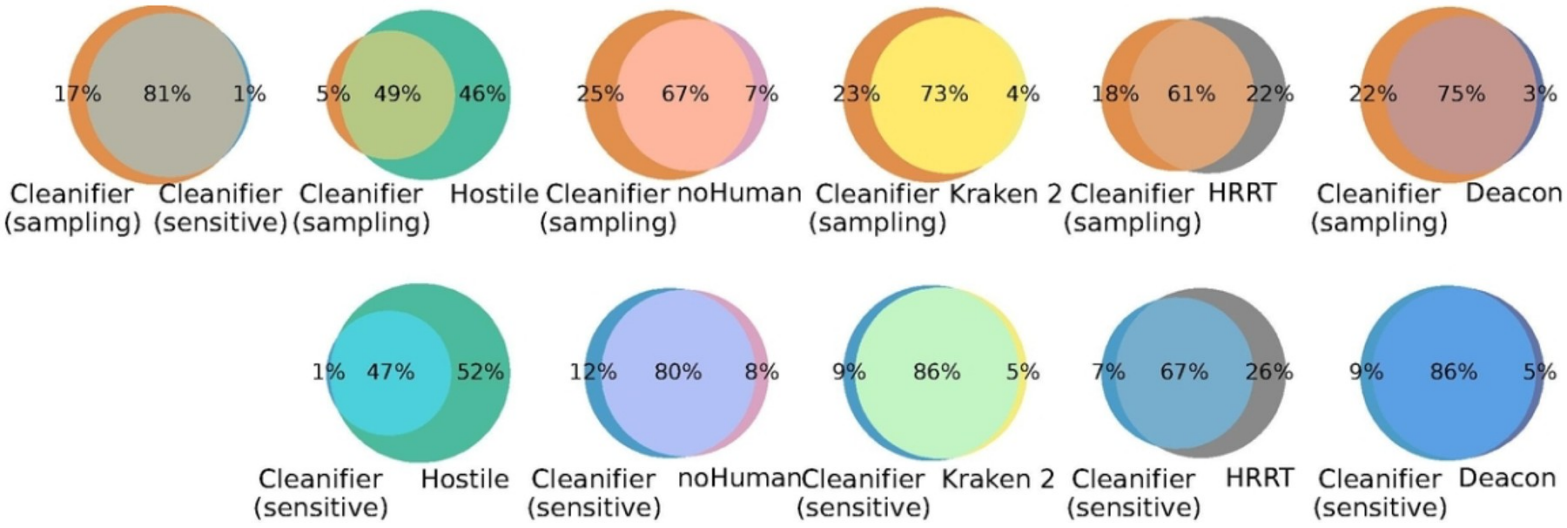
Overlap between retained human reads of HG002 between Cleanifier (probabilistic) and the other tools.

On both human and microbiome data, Cleanifier (sampling) is fastest, while the sensitive mode is slower than both Cleanifier (sampling) and noHuman, which is the second-fastest tool, almost on par with Deacon. The next fastest tools overall are Cleanifier (probabilistic, sensitive), HRRT, and Cleanifier (exact, sensitive). All of them are relatively slower on microbiome data than on human data, which can be explained by the fact that all *k*-mer lookups fail in the hash table. HRRT suffers more from this than the two sensitive Cleanifier versions.

Taxonomic classification with Kraken 2 using the standard database is slower compared to the smaller database used in noHuman. The overall running time of Kraken 2 + Krakentools is significantly higher, due to the slow filtering with Krakentools. In particular, Krakentools is very slow if large FASTQ files have to be written, as is the case for the microbiome data. In the case of human data, almost all data are discarded, resulting in a lower overhead of Krakentools.

Hostile is slowest on human data (72 times slower than the fastest method) due to the computationally expensive alignment. Perhaps surprisingly, it is among the fastest tools on microbiome data. The reason is that no alignment needs to be constructed for most of the reads since no start seed (short exact match) is found to initiate an alignment. In practice, human contamination in microbiome samples can reach up to 90% (Gao *et al.* 2025), requiring tools that are fast for classifying both human and non-human reads, which is achieved by Cleanifier.

### 3.5 Results on long reads

**Figure 4.**
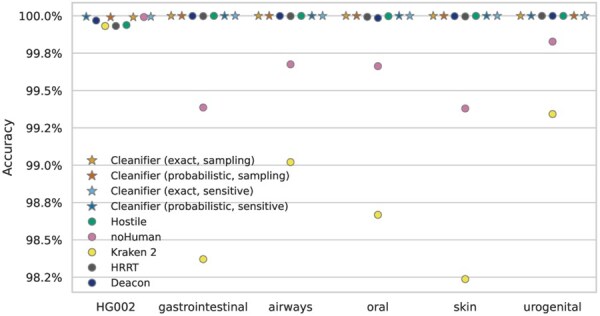
Accuracy, i.e. fraction of removed reads (human dataset HG002) or fraction of retained reads (microbiome datasets) for PacBio long reads; higher is better.

Figure 4 shows the accuracy on long reads. All tools, except Kraken 2 and noHuman, have an accuracy of almost 100% on the microbiome datasets (as for short reads, note the different *y*-axis scale in Figs 1 and 4). The lower accuracy of Kraken 2 and noHuman on long reads may be due to the probabilistic data structure used in Kraken 2. If more *k*-mers are queried, the probability of a false positive hit increases. This effect is less severe for Cleanifier (probabilistic) because Cleanifier classifies reads based on the percentage of covered bases, whereas Kraken 2 classifies the reads based on *k*-mer hits. The accuracy of all tools on the human HG002 dataset is >99.9%, with that of Deacon being below the Cleanifier variants and noHuman but above Kraken 2, HRRT and Hostile (note that this is a completely different dataset than the HG002 short read dataset, with different library preparation steps and likely without contamination due to filtering by read length). The higher accuracy compared to short reads results from the larger number of *k*-mers that together provide a stronger signal.

### 3.6 Running time on long reads

**Figure 5.**
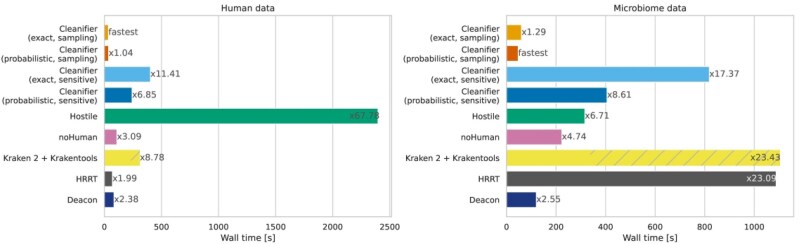
Wall clock running time in seconds for filtering 5 million long reads with eight threads (left panel: human data; right panel: microbiome data). Times for microbiome data are averaged over the five samples. Factors next to bars show running times relative to the fastest tool.

The relative wall clock running time between the tools on long reads is similar to the time on short reads (see Fig. 5). The sampling versions of Cleanifier are fastest on both types of datasets. On human data, the running times of HRRT, Deacon and noHuman are slightly slower (2–3 times), the sensitive mode of Cleanifier and Kraken 2 are significantly slower (8–12 times), and Hostile is extremely slow (68 times slower) because of the expensive alignments. On microbiome data, again Deacon and noHuman are slightly slower than the fastest tool (2.5–5 times), whereas the running time of HRRT, Kraken 2, and the sensitive exact version of Cleanifier increases sharply (18–24 times). In contrast, the running time of Hostile decreases and becomes comparable to the sensitive mode of Cleanifier (probabilistic). In summary, the sampling versions of Cleanifier, Deacon, and noHuman show consistently fast running times in all situations (see Fig. 5).

### 3.7 Memory requirements

Table 2 shows the memory requirements measured for filtering short read data. The memory is mostly used for holding the index data structure in memory, and to a lesser degree for sequence buffers. Hostile, HRRT, and Deacon have the smallest memory footprints below 5 GB. Between 5 and 7 GB is achieved by noHuman and Cleanifier (using the probabilistic Cuckoo filter with an FPR of $2^{-14}$). The exact representation of Cleanifier requires still under 16 GB, whereas the comprehensive Kraken 2 database needs much more memory, close to 90 GB. However, as seen in the case of noHuman, a smaller Kraken 2 database suffices to get a high accuracy.

**Table 2.** Maximum memory usage in GB; measured on short read samples.

	Cleanifier		Hostile	noHuman	Kraken 2	HRRT	Deacon
	exact	prob.					
Human	13.85	6.90	3.86	5.22	88.83	4.37	4.72
Microbiome	13.85	6.90	3.65	5.38	90.46	1.00	4.73

## 4 Conclusion and discussion

We presented a new tool, Cleanifier, for removing human (or other host) contamination from microbiome sequencing projects. Cleanifier is very fast in sampling mode, memory-efficient (it runs on an 8 GB system using the probabilistic Cuckoo filters) and offers consistently high accuracy on both short and long read data. In addition, Cleanifier supports building custom databases for other host organisms, e.g. for removing mouse reads from mouse gut microbiome samples.

The comparison between Cleanifier using an exact Cuckoo hash table and using a probabilistic Cuckoo filter shows that under the same circumstances, i.e. using the same references to build the index and applying the same classification method, the small FPR incurred by the probabilistic Cuckoo filter does not negatively affect the accuracy on short or long reads. However, results on very short reads (such as 50 bp reads sometimes used for transcript quantification) may be different, when a single gapped *k*-mer hit may decide about the classification.

The comparison between different tools for contamination removal showed that overall, Cleanifier and Deacon provide comparably high accuracy and speed; exact benchmark results will vary on different systems with different hardware properties. Interestingly, the design choices between Deacon and Cleanifier (exact minimizer sampling for Deacon; probabilistic storage of the complete gapped *k*-mer set, with optional sampling from the reads, for Cleanifier) are quite different. More detailed comparisons, including attempts to generate adversarial datasets for each tool, may lead to further improvements in the future.

## Supplementary Material

btaf632_Supplementary_Data

## Data Availability

All data used in the evaluation is available online (Zook *et al.* 2016, Meyer *et al.* 2025). Dataset URLs are provided in the Supplement, available as supplementary data at *Bioinformatics* online. The pre-built Cleanifier index is available at Zenodo (https://doi.org/10.5281/zenodo.15639519) or can be downloaded using the cleanifier download command.

## Code availability

The code and Snakemake workflows (Mölder *et al.* 2021) to reproduce all results are available via GitLab (https://gitlab.com/rahmannlab/cleanifier). In addition, Cleanifier is available at PyPi (https://pypi.org/project/cleanifier/) and Bioconda (https://anaconda.org/bioconda/cleanifier). An archived version of the implementation is available via Zenodo at https://doi.org/10.5281/zenodo.17286455.
