# Supplementary material for: Cleanifier: contamination removal from microbial sequences using spaced seeds of a human pangenome index
Source: Bioinformatics. 2025 Nov 18;42(1):btaf632. doi: 10.1093/bioinformatics/btaf632 (PMC12758600; doi:10.1093/bioinformatics/btaf632)
Supplement: btaf632_Supplementary_Data [file btaf632_supplementary_data.pdf]

Supplementary Material

Cleanifier: Contamination removal from microbial sequences using spaced seeds of a human pangenome index

Jens Zentgraf, Johanna Elena Schmitz and Sven Rahmann

Dataset URLs

	Short reads	Long reads
HG002	ftp://ftp-trace.ncbi.nlm.nih.gov/ReferenceSamples/giab/data/AshkenazimTrio/HG002_NA24385_son/NIST_Stanford_Illumina_6kb_matepair/fastqs/MPHG002-23100077/MPHG002_S1_L001_R1_001.fastq.gz, ftp://ftp-trace.ncbi.nlm.nih.gov/ReferenceSamples/giab/data/AshkenazimTrio/HG002_NA24385_son/NIST_Stanford_Illumina_6kb_matepair/fastqs/MPHG002-23100077/MPHG002_S1_L001_R2_001.fastq.gz	https://ftp-trace.ncbi.nlm.nih.gov/ReferenceSamples/giab/data_indexes/AshkenazimTrio/sequence.index.AJtrio_PacBio_CCS_10kb_09032018.HG002 sample IDs: 54238_180628_014238, 54238_180629_191119, 54238_180630_152934, 54238_180701_114913, 54315_180629_192151
HG003	ftp://ftp-trace.ncbi.nlm.nih.gov/ReferenceSamples/giab/data/AshkenazimTrio/HG003_NA24149_father/NIST_Stanford_Illumina_6kb_matepair/fastqs/MPHG003-23100078/MPHG003_S2_L002_R1_001.fastq.gz, ftp://ftp-trace.ncbi.nlm.nih.gov/ReferenceSamples/giab/data/AshkenazimTrio/HG003_NA24149_father/NIST_Stanford_Illumina_6kb_matepair/fastqs/MPHG003-23100078/MPHG003_S2_L002_R2_001.fastq.gz	
HG004	ftp://ftp-trace.ncbi.nlm.nih.gov/ReferenceSamples/giab/data/AshkenazimTrio/HG004_NA24143_mother/NIST_Stanford_Illumina_6kb_matepair/fastqs/MPHG004-23100079/MPHG004_S3_L003_R1_001.fastq.gz, ftp://ftp-trace.ncbi.nlm.nih.gov/ReferenceSamples/giab/data/AshkenazimTrio/HG004_NA24143_mother/NIST_Stanford_Illumina_6kb_matepair/fastqs/MPHG004-23100079/MPHG004_S3_L003_R2_001.fastq.gz	

	Short reads	Long reads
HG005	ftp://ftp-trace.ncbi.nih.gov/ReferenceSamples/giab/data/ChineseTrio/HG005_NA24631_son/NIST_Stanford_Illumina_6kb_matepair/fastqs/MPHG005-23100080/MPHG005_S4_L004_R1_001.fastq.gz, ftp://ftp-trace.ncbi.nih.gov/ReferenceSamples/giab/data/ChineseTrio/HG005_NA24631_son/NIST_Stanford_Illumina_6kb_matepair/fastqs/MPHG005-23100080/MPHG005_S4_L004_R2_001.fastq.gz	
HG006	ftp://ftp-trace.ncbi.nih.gov/ReferenceSamples/giab/data/ChineseTrio/HG006_NA24694-huCA017E_father/NIST_Stanford_Illumina_6kb_matepair/fastqs/MPHG006-23100081/MPHG006_S5_L005_R1_001.fastq.gz, ftp://ftp-trace.ncbi.nih.gov/ReferenceSamples/giab/data/ChineseTrio/HG006_NA24694-huCA017E_father/NIST_Stanford_Illumina_6kb_matepair/fastqs/MPHG006-23100081/MPHG006_S5_L005_R2_001.fastq.gz	
Gastrointestinal	https://frl.publisso.de/data/frl:6425518/gastrooral/<br>sample IDs: 0, 1, 2, 3	https://frl.publisso.de/data/frl:6425518/gastrooral_pbsim/<br>sample IDs: 0, 1, 2, 3
Oral	https://frl.publisso.de/data/frl:6425518/gastrooral/<br>sample IDs: 6, 7, 8, 13	https://frl.publisso.de/data/frl:6425518/gastrooral_pbsim/<br>sample IDs: 6, 7, 8, 13
Airways	https://frl.publisso.de/data/frl:6425518/airskinurogenital/<br>sample IDs: 7, 8, 10, 11	https://frl.publisso.de/data/frl:6425518/airskinurogenital_pbsim/<br>sample IDs: 7, 8, 10, 11
Skin	https://frl.publisso.de/data/frl:6425518/airskinurogenital/<br>sample IDs: 1, 13, 14, 15	https://frl.publisso.de/data/frl:6425518/airskinurogenital_pbsim/<br>sample IDs: 1, 13, 14, 15
Urogenital	https://frl.publisso.de/data/frl:6425518/airskinurogenital/<br>sample IDs: 0, 2, 3, 6	https://frl.publisso.de/data/frl:6425518/airskinurogenital_pbsim/<br>sample IDs: 0, 2, 3, 6

Table 1: Data URLs for human (GIAB) and microbiome (CAMI 2 challenge) data. The data is subsampled (for human) or combined (for microbiome) such that each file contains 50 million short reads or 5 million long reads.

Cleanifier speedup factors

Figure 1 shows the speedup for parallelization, which is almost linear for up to 8 classifier threads, except for the probabilistic sampling version. For Cleanifier (probabilistic, sampling), the read or write thread becomes the main bottleneck when more than five threads perform the classification.

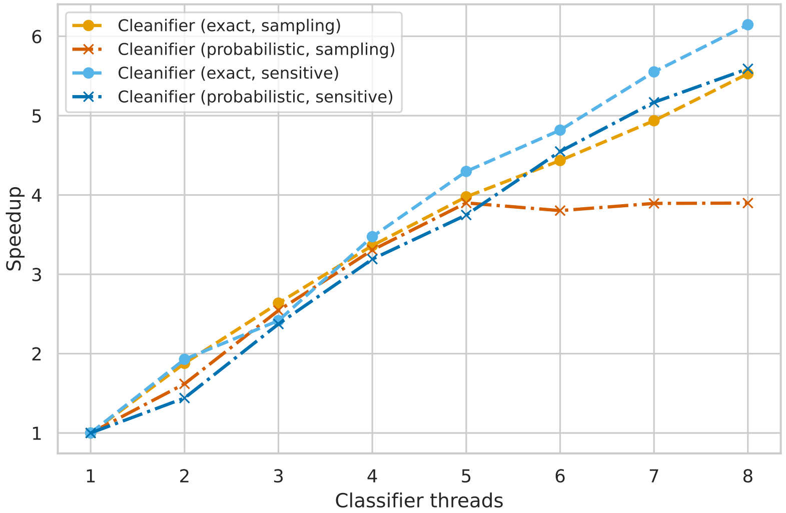

Figure 1: Speedup for increasing classifier threads. Total threads are the number of classifier threads plus one additional read and one additional write thread.

Accuracy for different classification thresholds and k-mer masks

We built indexes for 6 different gapped k-mer masks that were selected from the best performing gapped k-mer masks evaluated in [5]; see Table 2 for the list of masks and their shapes.

Figure 2 shows the accuracy for human and microbiome data for the different masks and different classification thresholds. Due to the good performance on both human and microbiome data, we selected the (29,33)-mask for the pre-built human pangenome index and set the default threshold for classification to 0.5.

k	w	mask
23	33	`####_##__#_####_#_####_#__##_####`
27	31	`######_#####_#####_#####_######`
29	33	`######_#######_###_#######_######`
29	37	`####_####_###_###_#_###_###_####_####`
31	37	`######_###_####_#####_####_###_######`
31	43	`####_#_##_###_#_###_###_###_#_###_##_#_####`

Table 2: Evaluated gapped k-mer masks.

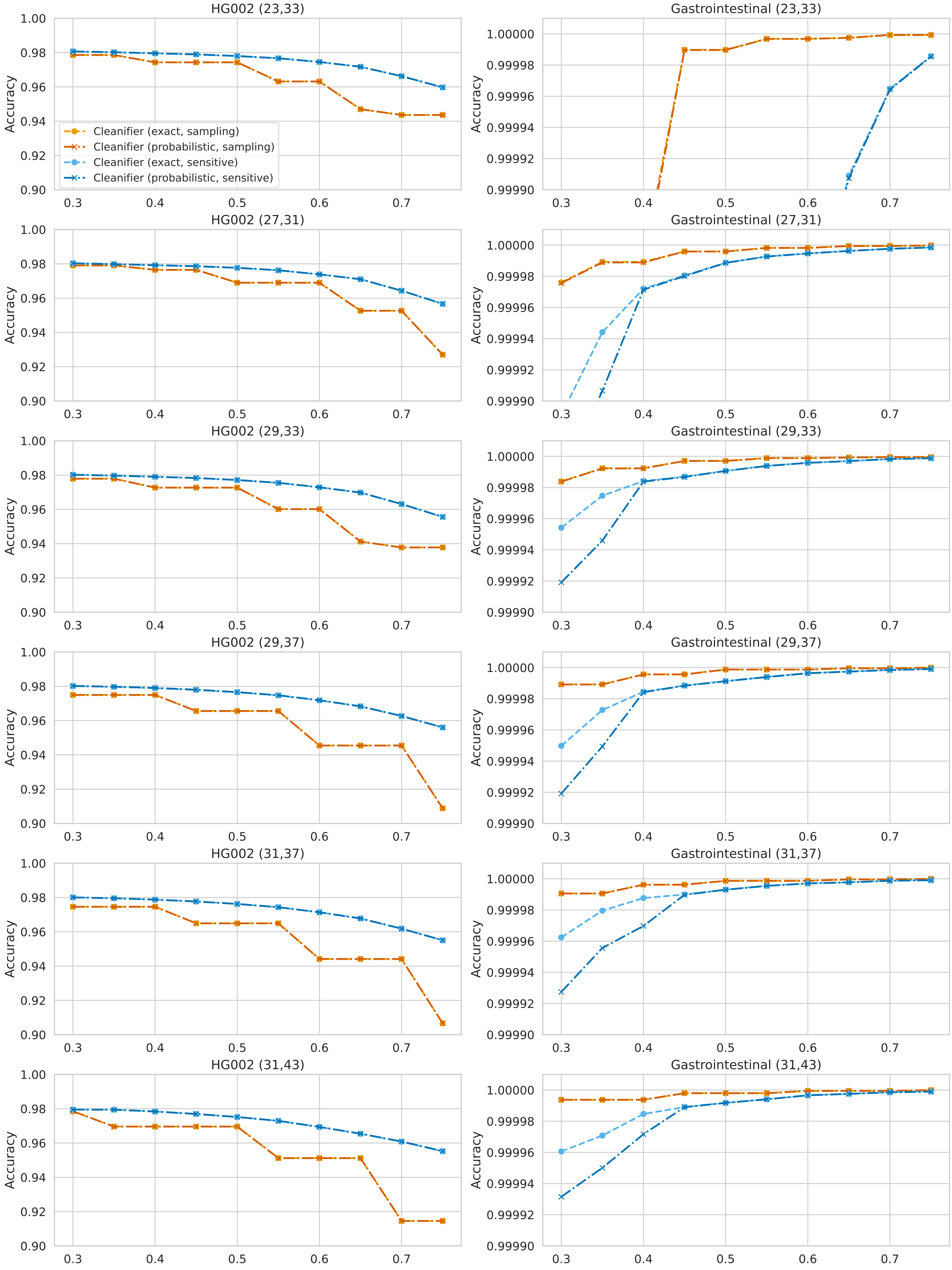

Figure 2: Accuracy for human and microbiome data for different indexes (built using different gapped k-mer masks) and different classification thresholds.

Comparison of a T2T index and the human pangenome index

Table 3 shows the accuracy for an index containing only the gapped k-mers from the T2T reference [4] and from our advanced human index that is built from the T2T reference, the Ensenbl cDNA file, the common variants from the 1000 Genome Project [1], the HLA variants from the IPD-IMGT/HLA database [2] and the 47 assemblies from the Human Pangenome Consoritium [3]. Our advanced index leads to a higher accuracy on human data both for the exact and probabilistic version, with a higher increase in the accuracy for the sampling mode. The microbiome retention is only marginally reduced by the more comprehensive human pangenome index.

	Cleanifier exact				Cleanifier probabilistic			
	sampling		sensitive		sampling		sensitive	
	T2T	pangenome	T2T	pangenome	T2T	pangenome	T2T	pangenome
Human	0.969986	0.972677	0.976269	0.977091	0.969988	0.972680	0.976280	0.977103
Microbiome	0.999999	0.999997	0.999994	0.999991	0.999999	0.999997	0.999994	0.999991

Table 3: Accuracy for an index built for the T2T reference and for an index containing all gapped k-mers of the T2T reference, cDNA, common variants, HLA variants and multiple pangenome assemblies. We measured the accuracy on the HG002 and gastrointestinal short read datasets using the default classification threshold of 0.5.

Pairwise overlap between retained human reads

Figure 3 shows the pairwise overlap of the sets of reads that are retained in the HG002 dataset by all evaluated tools. Since all tools have a high overlap, it is possible that many of these reads are either contaminants, like PhiX or the Eppstein-Barr virus, or poor quality reads and should indeed be retained as non-human.

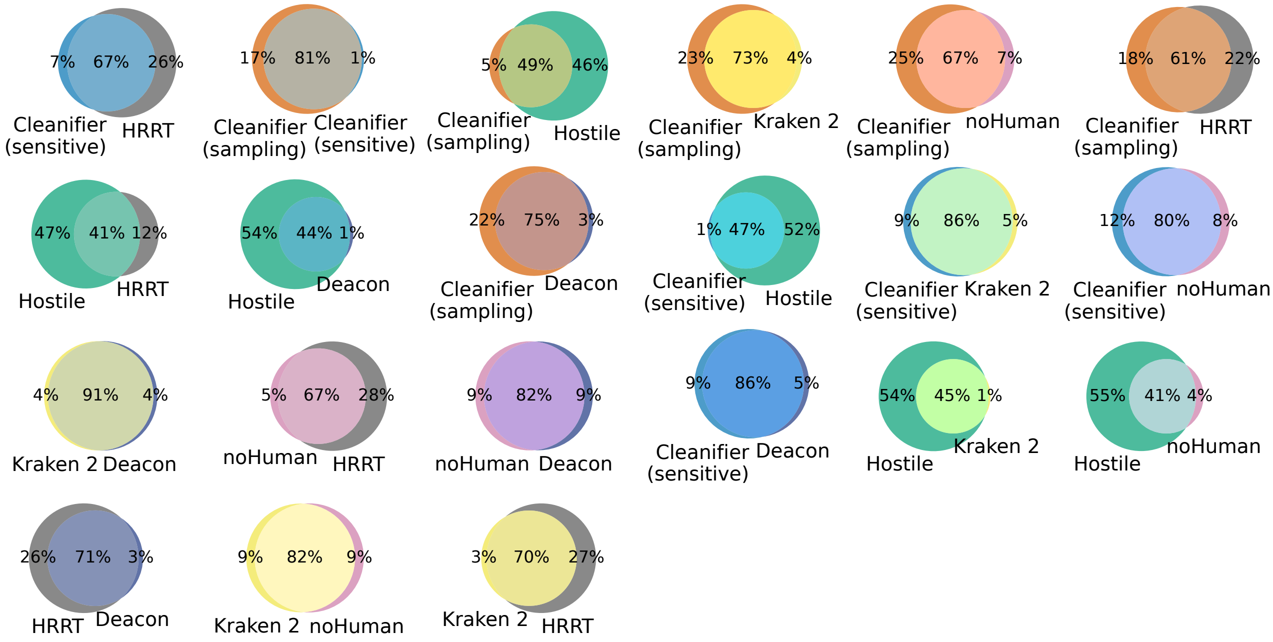

Figure 3: Overlap of retained reads for the HG002 dataset between all tools. We only included Cleanifier (probabilisitc) in the comparison.

Comparison between tools using the T2T human reference

Figures 4, 5 and 6 show the performance in terms of accuracy, running time and memory requirements using a human index that contains only the T2T human reference genome for all tools. This allows us to evaluate whether the performance differences between the tools are due to different indexes or algorithmic design. All other parameters and data is the same as in the main article (Figures 2-3, Table 2). We exclude HRRT and noHuman because HRRT does not support building a custom index and noHuman is a Kraken 2 wrapper with a fixed custom database.

Figure 4 shows that Cleanifier and Deacon falsely keep more human reads and have better microbiome retention when using the T2T index compared to the pre-built indexes. A comparison of the accuracy for using the different indexes in Cleanifier is also given in Table 3. The microbiome retention of Kraken 2 is considerably lower for the T2T index. The accuracy of Hostile is almost identical for both indexes.

For all tools, using a different index has only a small impact on the running time of the filtering step.

As expected, Cleanifier's maximum memory usage (and Hostile's to a lesser extent) is smaller than when using the human pangenome index. The maximum memory usage of Kraken 2 is much lower (≈ 90 vs. 5 GB), which is comparable to the human index used in noHuman (5.4 GB). The memory usage of Deacon is almost identical for both indexes, because the pre-built indexes is built for more human references but excludes some k-mers that are shared between humans and bacteria or viruses. Deacon requires less space than Cleanifier (for both the exact and probabilistic version) because it stores only the minimizers, whereas Cleanifier indexes all gapped k-mers and performs sampling at the read level (and does not sample in sensitive mode).

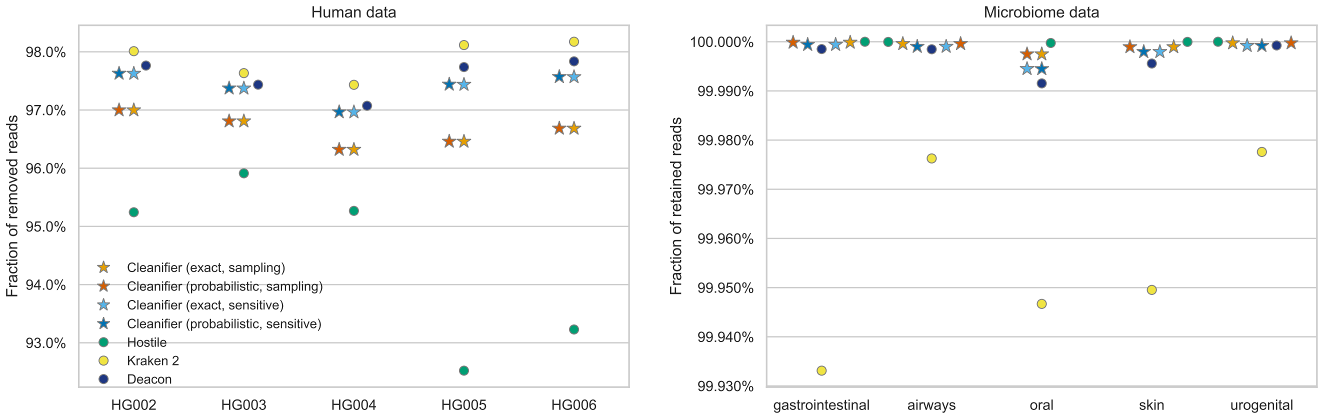

Figure 4: Accuracy for short reads using an index that contains for all tools only the T2T human reference genome. The data is the same as in Figure 2.

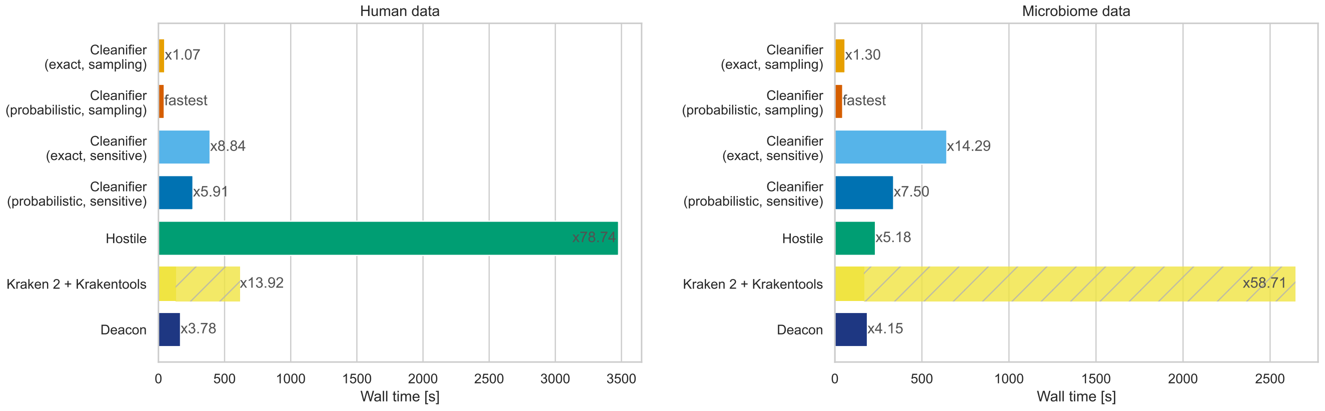

Figure 5: Wall clock running time for using the custom T2T index for all tools. Apart from the different index, the data and parameters used are the same as in Figure 3.

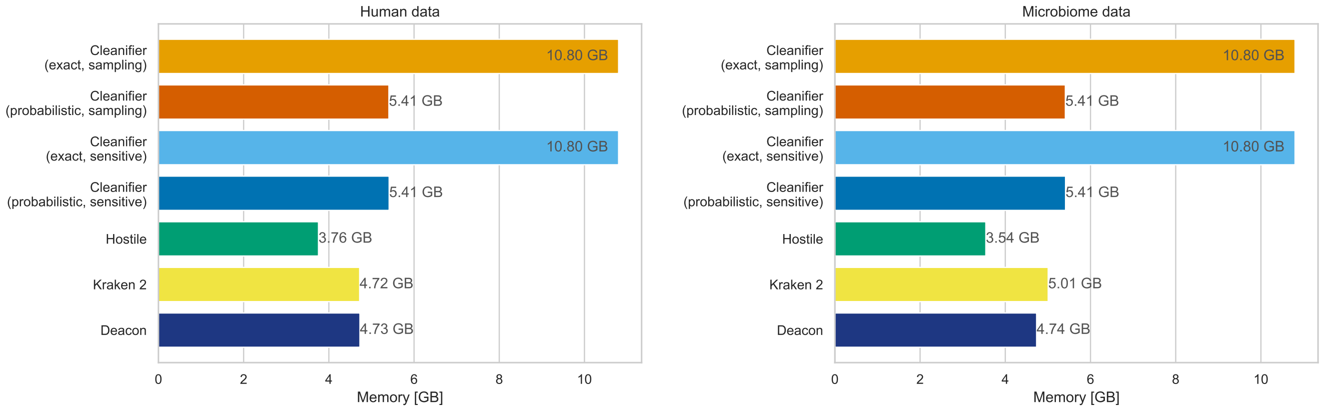

Figure 6: Maximum memory usage in GB for T2T indexes. The index size of the k-mer-based tools depend on k, whether the index contains all k-mers (Cleanifier) or if a minimizer index is built (Deacon, Kraken 2), and whether an exact data structure (Cleanifier (exact), Deacon) or a probabilistic datastructure is used (Cleanifier (probabilistic), Kraken 2).

References

[1] Adam Auton et al. A global reference for human genetic variation. *Nature*, 526(7571):68–74, 2015.

[2] Dominic J Barker et al. The IPD-IMGT/HLA database. *Nucleic Acids Research*, 51:D1053–D1060, 2023.

[3] Wen-Wei Liao et al. A draft human pangenome reference. *Nature*, 617(7960):312–324, 2023.

[4] Sergey Nurk et al. The complete sequence of a human genome. *Science*, 376(6588):44–53, 2022.

[5] Jens Zentgraf and Sven Rahmann. Design of worst-case-optimal spaced seeds. In Broňa Brejová and Rob Patro, editors, *25th International Conference on Algorithms for Bioinformatics (WABI 2025)*, volume 344 of *Leibniz International Proceedings in Informatics (LIPIcs)*, pages 22:1–22:17. Schloss Dagstuhl – Leibniz-Zentrum für Informatik, 2025.
